# Long-afterglow metal–organic frameworks: reversible guest-induced phosphorescence tunability†

**DOI:** 10.1039/c6sc00563b

**Published:** 2016-03-24

**Authors:** Xiaogang Yang, Dongpeng Yan

**Affiliations:** a Key Laboratory of Theoretical and Computational Photochemistry, Ministry of Education Beijing Normal University Beijing 100875 P. R. China; b State Key Laboratory of Chemical Resource Engineering Beijing University of Chemical Technology Box 98 Beijing 100029 P. R. China yandp@bnu.edu.cn +86 10-6442-5385

## Abstract

Luminescent metal–organic frameworks (MOFs) have received much attention due to their wide structural tunability and potential application in light-emitting diodes, biological imaging and chemical sensors. However, successful examples of long-persistent afterglow MOFs are still quite limited to date. In this work, we report that two types of Zn-terephthalate (TPA) MOFs (namely [Zn(TPA)(DMF)] (1-DMF) and MOF-5) could exhibit an obvious room-temperature afterglow emission with a time-resolved luminescence lifetime as high as 0.47 seconds. The phosphorescence-based afterglow was also highly sensitive to the temperature, and the reversible emission intensity could be recycled under high/low temperatures. Moreover, both 1-DMF and MOF-5 showed highly tunable afterglow phosphorescence colors (from cyan to yellow and from green to red, respectively) upon treatment with pyridine solution. The fluorescence/phosphorescence emission color of MOF-5 can be reversibly switched due to the addition and removal of a pyridine guest to and from the host nanochannel, as shown in both experimental and computational studies. Therefore, this work not only shows a facile method to develop MOF-based long-afterglow materials at room temperature, but also presents a strategy to tune their phosphorescence in a wide range based on host–guest interactions.

## Abstract 2

We report that two types of Zn-terephthalate (TPA) MOFs (namely [Zn(TPA)(DMF)] (1-DMF) and MOF-5) could exhibit an obvious room-temperature afterglow emission with a time-resolved luminescence lifetime as high as 0.47 seconds.

## Graphical abstract

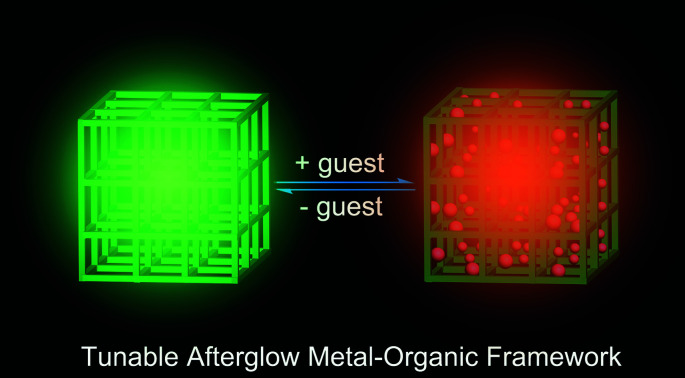

## Introduction

Afterglow, long-lasting phosphorescence and persistent luminescence, which can last for an appreciable time after the removal of the excitation source, have attracted great attention during the last few decades,^1^ owing to their commercial applications in the fields of traffic signs, interior decoration, emergent-lighting and displays,^2^ as well as their potential use in chemical sensors, optical recording devices, biological imaging, and security systems.^3^ Since Matsuzawa *et al.* reported the long-lasting afterglow of SrAl_2_O_4_:Eu^2+^ co-doped with Dy^3+^ ions in 1996,^4^ research on efficient persistent phosphors has continuously gained much interest. However, the number and type of persistent luminescent materials are still relatively limited to date, and the interest worldwide has mainly focused on rare-earth (RE)-containing inorganic materials.^5^ Unfortunately, the high-cost and relatively complicated preparation methods (such as high temperature solid-state processes) present a barrier to commercialization.^6^ Therefore, the development of new types of inexpensive, energy-efficient, eco-friendly, and RE-free afterglow materials is highly desirable.

With their combination of inorganic units and organic links, metal–organic frameworks (MOFs) have received much attention recently in both fundamental research and industrial fields for their applications in gas storage/separation,^7^ catalysis,^8^ drug delivery,^9^ and energy storage and conversion.^10^ Moreover, luminescent MOFs have also been extensively studied owing to their facile synthesis routes, tunable structures and applications in light-emitting diodes,^11^ biological imaging^12^ and chemical sensors.^13^ Luminescent MOFs^14^ containing noble-metal (NM, such as Pt, Ir and Ru) and RE (such as Eu, Tb) usually present photoemission lifetimes in the range of hundreds of microseconds (μs) to a few milliseconds (ms) based on the ligand–metal or metal–ligand charge transfer (LMCT/MLCT) mechanism. However, the relatively low luminescence decay time cannot meet the requirements of persistent luminescence applications, since the recognition of afterglow emission for the common human generally requires the emission decay at the tens of ms level. Recently, Sun *et al.* have reported that a Cd-based MOF could exhibit afterglow behavior only under low-temperature (77 K) conditions;^15^ therefore, obtaining long-afterglow phosphors under ambient conditions based on the facile design of MOFs continues to be a challenging goal.

**Scheme 1:**
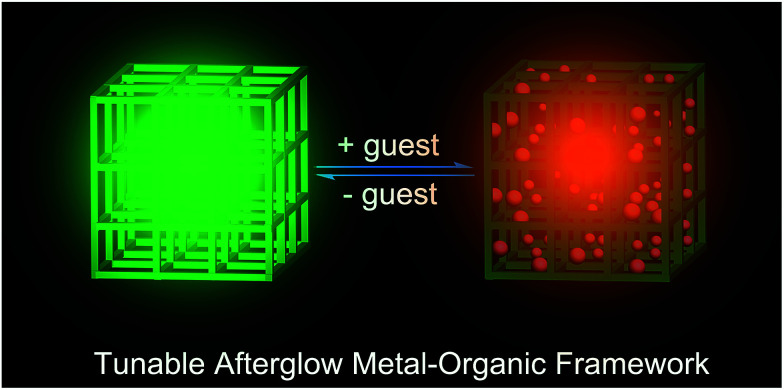
Schematic representation of the tunable afterglow MOFs based on host–guest interactions.

On the other hand, MOFs with dynamic frameworks can reversibly change their structures and significantly improve their desirable properties for smart material applications.^16^ Typically, dynamic MOFs involve a guest-induced transformation between two or more structures upon solvation and/or guest adsorption.^17^ In some cases, the guest-induced structural change can result in a sensory response (such as fluorescence^18^ or magnetism^19^). Although much effort has been devoted to these dynamic MOF-based materials,^20^ host–guest systems combining both tunable long-lasting afterglow and a smart MOF sensor have rarely been reported (Scheme 1). Herein, we report that two types of Zn-terephthalate (TPA) MOFs (namely [Zn(TPA)(DMF)] (1-DMF) and MOF-5) could exhibit an obvious room-temperature afterglow emission with a time-resolved luminescence lifetime as high as 0.47 seconds. Moreover, the as-obtained MOF samples could present highly tunable phosphorescence colors (from cyan to yellow and from green to red, respectively) based on the introduction of guest molecules into the MOF nanochannels. Periodic density functional theory calculations showed that the guest molecules can largely regulate the frontier orbitals and electronic structures of the pristine MOF host structures. Therefore, this work supplies a cost-effective way to obtain high-performance long-afterglow MOFs at room temperature, which can further serve as potential heat and solvent phosphorescence sensors.

## Results and discussion

**Fig. 1:**
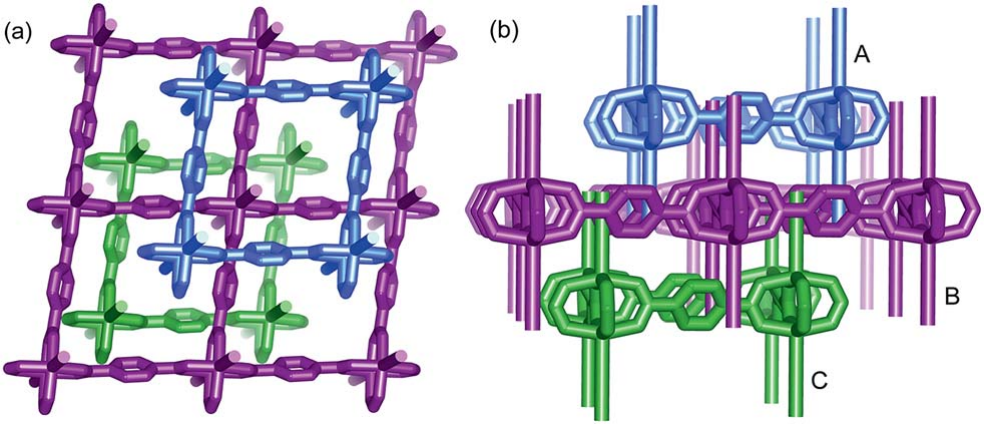
View of the 2D → 3D polythreading architecture from [111] (a) and [010] direction (b). Two dangling DMF pendants (simplified by sticks for clarity), from the adjacent layers above and below (A and C), thread each window of the central one (B).

TPA is one of the representative aromatic ligands, and the selection of TPA as the organic link is based on the expectation that the potential n → π* transition of the carboxylic or carbonyl group is beneficial to the enhancement of spin–orbit coupling and long-lived triplet excited states after it is fixed in the rigid MOFs. Through a solvothermal process, Zn(NO_3_)_2_·6H_2_O and TPA (2 : 1 molar ratio) were dissolved in *N*,*N*′-dimethylformamide (DMF) and heated at 100 °C for 6 h, and then the colorless block-shaped crystals of [Zn(TPA)(DMF)] (1-DMF) were formed (Fig. S1a in ESI†). A longer solvothermal time gave a product with poor crystallinity, which may be due to an increase in the pH value upon the production of amine from the decomposition of DMF.^21^ Additionally, when the same reaction mixture was heated at 120 °C for 6 h, cubic block crystals of MOF-5 were obtained (Fig. S1b†). X-ray single crystal structural analysis reveals that 1-DMF crystallizes in a monoclinic *C*2/*m* space group and shows 2D → 3D polythreading architecture. In the structure of 1-DMF, paddle-wheel dinuclear zinc carboxylate secondary building units (SBUs) {Zn_2_(COO)_4_} with a Zn⋯Zn distance of 2.949 Å are bridged by the TPA ligands in a di-monodentate fashion to form a 2D microporous layer. The oxygen atoms coordinated in the DMF molecules occupy the axial sites of the {Zn_2_(COO)_4_} paddle wheel, hanging on two sides of each layer (Fig. S2†). These layers are stacked in an offset fashion with an ABCABC sequence, resulting in a 3D framework (Fig. S1c†). Notably, each window of the same layer is threaded by two dangling DMF pendants, one each from the adjacent layers above and below, to generate a 2D → 3D polythreading architecture (Fig. 1). Although stacked in an offset fashion, the 3D framework of 1-DMF exhibited 1D channels (8.7 × 8.7 Å^2^) containing coordinated DMF molecules along the [001] direction.

**Fig. 2:**
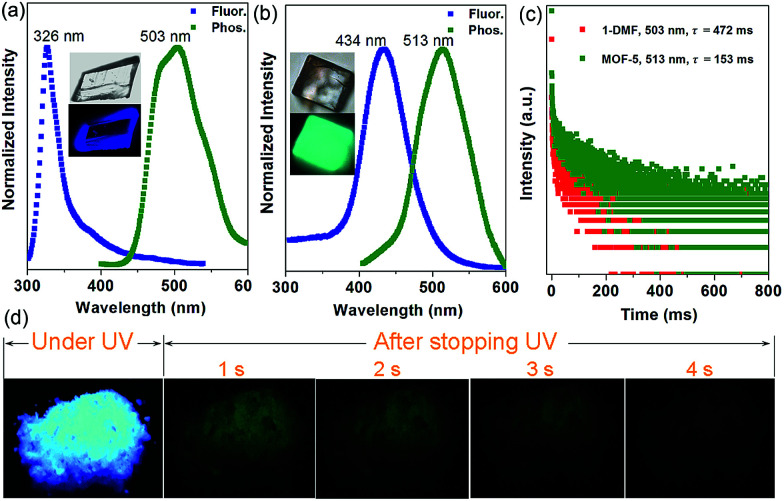
Normalized photoluminescence spectra for 1-DMF (a) and MOF-5 (b) with excitation at 280 and 330 nm, respectively. (c) Time-resolved phosphorescence decay curves of 1-DMF and MOF-5. (d) Photographs of the afterglow of 1-DMF taken at different time intervals before and after turning off the UV excitation (365 nm) under ambient conditions. Photographs of the crystals under daylight and UV light are shown in the insets.

Interestingly, the crystalline 1-DMF and MOF-5 present fluorescence emission at 326 and 434 nm (Fig. 2a and b) with a fluorescence quantum yield of 2.25% and 46.27%, respectively. The corresponding crystal images under UV light and daylight are shown in the insets. Under ambient conditions, phosphorescence emissions at 503 and 513 nm for 1-DMF and MOF-5 were detected, with a phosphorescence quantum yield of 4.76% and 0.22%, respectively. The difference in photoemission properties can be ascribed to the different stacking fashions of the ligands and different Zn-based metal clusters between the two MOFs. In addition, the coordinated DMF in 1-DMF may also influence the photophysical process. Moreover, the phosphorescence decay curve gave their phosphorescence lifetime values as *ca.* 472 and 153 ms, respectively (Fig. 2c). Particularly, after the removal of the UV light, green emission could be recognized easily by the naked eye, and was also captured using a digital camera in the time range of 0–4 seconds, confirming the long-afterglow characteristics for the Zn-based MOFs (Fig. 2d).

Powder X-ray diffraction (PXRD) confirmed the high purity of both the 1-DMF and MOF-5 samples (Fig. S3†), and thus excluded any emission from pristine TPA crystals. It was reported that pure TPA presents very weak phosphorescence with an emission lifetime of *ca.* 0.5 ms,^22^ and thus the lifetime value has been enhanced by nearly three orders of magnitude upon the formation of MOF structures, relative to the pure ligands. Moreover, compared with the classical RE-doped afterglow phosphors (such as SrAl_2_O_4_:Eu^2+^/Dy^3+^) with the same emission color before and after turning off the UV source, the two MOF samples in this work present obviously changeable emissive colors due to the fluorescence/phosphorescence transformation. Such time-dependent afterglow changes may supply the MOF systems with an opportunity for potential optical antiforgery applications.

**Fig. 3:**
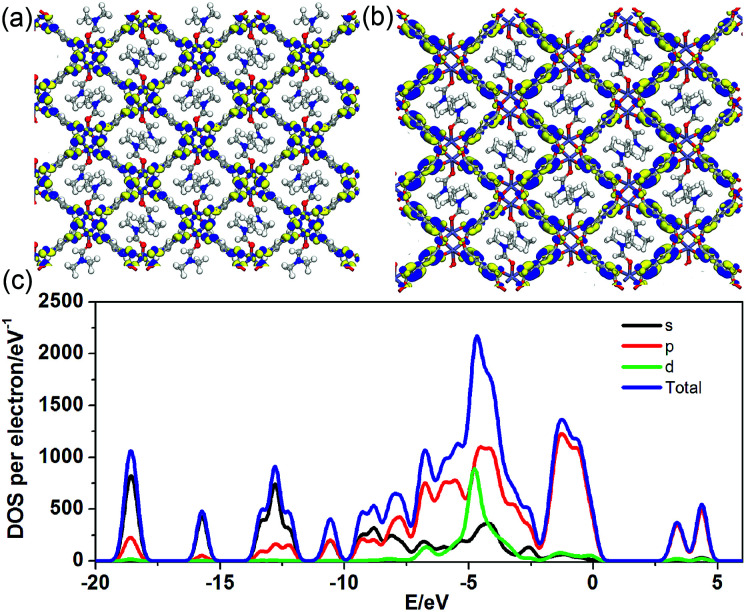
Frontier orbital (HOMO (a) and LUMO (b)) profiles and total/partial electronic density of state (TDOS and PDOS, (c)) for the DFT-optimized structure of 1-DMF (H: white; O: red; C: gray; Zn: blue-gray; N: blue).

To better understand the long-persistent afterglow as well as the electronic structure of 1-DMF, periodic density functional theory (PDFT) calculations were performed (Fig. 3 and S4†). Total/partial electronic density of state (TDOS and PDOS) showed that 1-DMF presents a low band gap (3.31 eV, 375 nm), which may correspond to the fluorescence emission around 326 nm. Around the Fermi level, the TDOS mainly consists of the 2p electrons of the O atoms from the TPA ligand and the d electrons from the Zn atoms. Frontier orbital analysis shows that the electron-density distribution of the highest occupied molecular orbital (HOMO) is mainly located on Zn_2_O_8_ metal nanoclusters containing Zn(ii) ions and O atoms in the TPA ligands, the lowest singlet excitation is governed by the transition from HOMO to the lowest unoccupied molecular orbital (LUMO). The electron-density distribution of the LUMO and LUMO+1 is dominated by TPA ligands. Therefore, the photo-excitation and emission involves an MLCT process from the Zn_2_O_8_ units to the organic links, which usually present a prolonged excitation lifetime. From the combination of both experimental and computational results, it can be concluded that the long-afterglow of the 1-DMF sample can be attributed to the following reasons: (1) the Zn(ii) ion has participated in the frontier orbital distributions and thus may promote spin–orbit coupling and improve the electron–hole separation time during the charge transfer process; (2) the molecular vibrations and nonradiative loss can be highly reduced based on the coordination interactions between Zn(ii) and TPA, since the organic ligands are tightly fixed and locked in the rigid framework. This may further increase the intersystem crossing between the S_1_ and T_1_ excited states.^23^

**Fig. 4:**
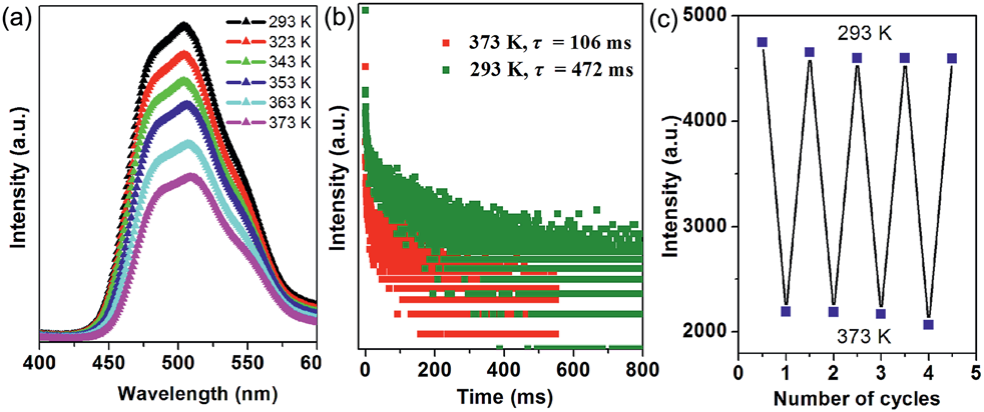
(a) Steady-state phosphorescence spectra of 1-DMF measured at temperatures from 293 to 373 K. (b) Time-resolved emission decay curves of 1-DMF crystals at 293 and 373 K. (c) Reversible variation of the phosphorescence intensity of 1-DMF at 293 and 373 K.

Thermal behavior is important to understand the coordination states for the MOF phosphors. Thermogravimetric analyses (Fig. S5a†) show that 1-DMF is stable up to about 130 °C, and the weight loss of 24.3% in the temperature range 130–330 °C corresponds to the release of coordinated DMF molecules (calc. 24.5%). Above 380 °C, 1-DMF begins to decompose due to the burning of the TPA units. In the case of MOF-5 (Fig. S5b†), the weight losses of the guest molecules are in the temperature range of 80 to 220 °C, and the resulting porous framework starts to decompose after 410 °C. Furthermore, to detect the heat-related persistent afterglow, temperature-dependent phosphorescence spectra of the 1-DMF crystals were further measured. It was observed that the bands at 503 nm were decreased obviously upon the increase of the temperature from ambient conditions (293 K) to 373 K (Fig. 4a). The corresponding phosphorescence lifetime also decreased to a value of 106 ms at 373 K (Fig. 4b). Similar to other long-afterglow materials, this behavior is related to the fact that the molecular vibrations and nonradiative loss greatly increased under high temperature. Moreover, if 1-DMF was cooled down to 293 K, it was observed that the phosphorescence emissive intensity can nearly recover its original position, and the reversible change in luminescence can be readily repeated at least 4 times during such heating–cooling cycles (Fig. 4c). Therefore, 1-DMF can potentially serve as a phosphorescence sensor for heat detection.

Open coordination sites (OCSs) often play a key role in functional MOFs, such as in gas separation/sorption and catalysis.^24^ Recently, it has been shown that Zn-based paddle wheel SBUs present a dynamic structural transformation upon the removal and rebinding of guest molecules.^17,21^ In the network of the 1-DMF of this work, large channels may allow the fast access of guests through their large open spaces. In addition, the coordinated DMF molecules are also exposed in the MOF nanochannels. With the development of a potential afterglow-based sensor in mind, the potential solvatochromic emission behavior of 1-DMF was detected by measuring the luminescence of the samples upon treatment with different solvents (Fig. S6†). It was observed that the phosphorescence quenching occurred to different extents after the solvent treatments. Specifically, the phosphorescence intensity of 1-DMF decreased obviously after being treated with MeOH and acetone, suggesting that 1-DMF may serve as a phosphorescent sensor for MeOH and acetone through phosphorescence quenching (Fig. S6†). This observation may be related to the substitution of the coordinated DMF by other solvents, which results in the alternation of the pristine crystal structure and related photophysical properties. This can be further confirmed by the change in the PXRD patterns before and after the solvent treatment (Fig. S7†). Meanwhile, the wavelength shifts of the phosphorescence emission have been observed after treatment with water and pyridine solvents. For example, after soaking the 1-DMF in water (1-Water), the sample undergoes a phosphorescence red-shift of *ca.* 6 nm; further the PXRD measurement showed that the 1-Water system presents an obvious structural difference compared with the 1-DMF. However, 1-Water exhibits an isostructuralism with MOF-2,^25^ indicating that a crystal-to-crystal transformation occurs during water treatment (Fig. S7 and Table S3†). Thus, the overall framework connectivity in 1-Water was maintained, while the replacement of the DMF ligand by water molecules shortened the nearest interlayer Zn⋯Zn separation from 6.44 to 4.83 Å, giving rise to a slight volume decrease. This is due to the smaller steric requirement of H_2_O compared to that of DMF. Meanwhile, the increase in the distance between two neighboring paddle-wheel units in the layer can also be observed (Fig. S8†).

**Fig. 5:**
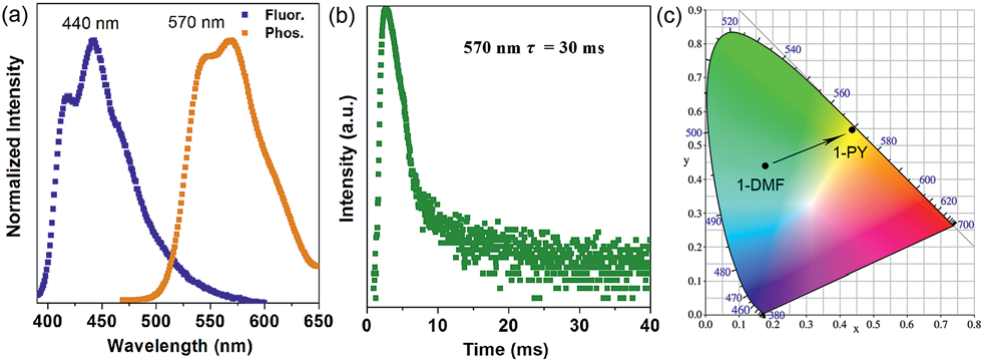
Normalized photoluminescence spectra (a) and time-resolved phosphorescence decay curves (b) of 1-PY, (c) corresponding CIE color coordinates for the phosphorescence of 1-DMF and 1-PY.

More interestingly, when 1-DMF was treated with pyridine (PY) to obtain a 1-PY solid sample, there are obvious luminescence changes in the fluorescence and phosphorescence located at 440 and 570 nm (*λ*_ex_ = 350 nm, Fig. 5a), with maximum wavelength shifts of 114 nm and 67 nm, respectively. The phosphorescence emission undergoes a remarkable transformation from a cyan (color coordination: (0.178, 0.440)) to yellow color (color coordination: (0.439, 0.549)) as shown in Fig. 5c, and the corresponding phosphorescence lifetime is also reduced to *ca.* 30 ms (Fig. 5b). The PXRD studies showed that the structure of 1-PY is also similar to MOF-2 (Fig. S7†), and thus it can be speculated that the coordinated DMF units have been replaced by the PY.

**Fig. 6:**
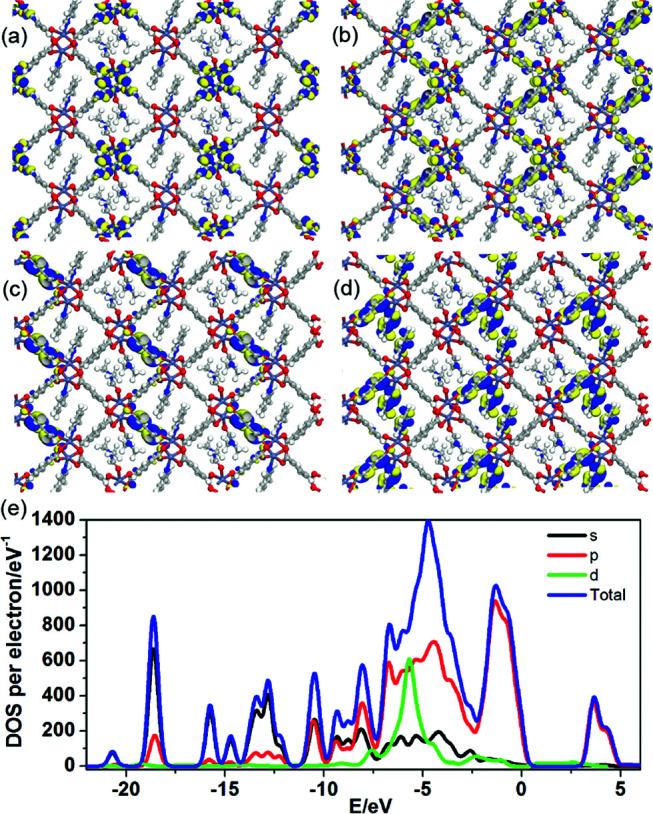
Frontier orbital (HOMO (a), HOMO−1 (b), LUMO (c) and LUMO+1 (d)) profiles and total/partial electronic density of state (TDOS and PDOS, (e)) for the DFT-optimized structure for 1-PY (H: white; O: red; C: gray; Zn: blue-gray; N: blue).

To better understand the notable emission shifts for 1-PY compared with those of 1-DMF, we further carried out PDFT calculations on the idealized 1-PY model, in which half of the DMF molecules have been substituted by coordinated PY (Fig. 6). Frontier orbital analysis shows that the electron-density of the HOMO and HOMO−1 is mainly located on the Zn_2_O_8_ metal cluster containing Zn(ii) ions and O atoms in the TPA ligands (Fig. 6a and b), while the electron-density distribution of the LUMO and LUMO+1 is mainly populated on both the TPA and PY (Fig. 6c and d). The residual DMF molecules have not contributed to the frontier orbital distribution. Moreover, band structure analysis showed that 1-PY presents a reduced band gap of 3.02 eV (*ca.* 411 nm), which could rationally correspond to the emission red-shift in the experiment. Therefore, it can be concluded that the coordinated PY has participated in the photoemission process, and the introduction of intermediate energy levels has further shortened the band gap, which could result in the wavelength red-shift for both fluorescence and phosphorescence. Furthermore, the occurrence of energy/electronic transfer from the MOF host to the PY guest could also result in a loss of triplet energy in the host framework, and thus leads to a reduced phosphorescence lifetime.

**Fig. 7:**
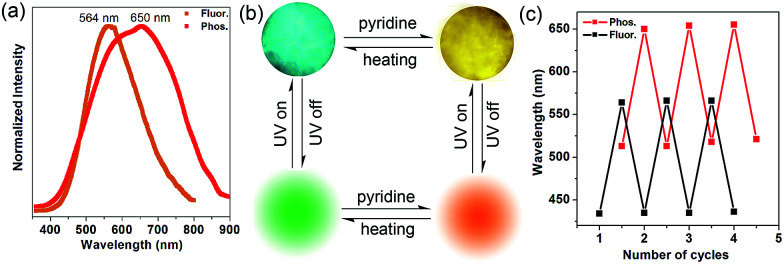
(a) Normalized photoluminescence spectra for MOF-5 after treatment with PY. (b) Top line: the MOF-5 crystal under fluorescence microscopy before and after treatment with pyridine; bottom line: schematic representation of the tunable phosphorescence under the heating and guest readsorption process. (c) Reversible variation of fluorescence and phosphorescence emission wavelengths between the desolvation and guest readsorption process of MOF-5.

Inspired by the highly tunable color of the phosphorescence from the 1-DMF to the 1-PY system, the MOF-5 crystal was further soaked with PY solvent. Upon excitation at 350 nm under ambient conditions, we observed that MOF-5 also underwent obvious luminescence changes with the fluorescence and phosphorescence emission located at 564 nm (yellow color) and 650 nm (red color), respectively (Fig. 7a). The PXRD profiles showed that the pattern of MOF-5 was nearly unchanged before and after treatment with PY, suggesting that the host framework was maintained (Fig. S9†). UV/vis spectra (Fig. S10†) show that the optical absorption characteristics of 1-DMF, MOF-5 and PY@1-DMF present very similar absorption positions below 310 nm. While for the PY@MOF-5 sample, two new absorption peaks (353 and 420 nm) appeared, which can be assigned to the introduction of intermediate energy levels from the PY guest molecules, as was also confirmed by the theoretical calculation below.

Considering that MOF-5 presents a relatively large channel (size: 1.1 nm × 1.1 nm) without additional coordination sites, the PY molecules can potentially be guests in the nanochannel based on π–π interactions with the TPA ligands. Therefore, the color-changed PY@MOF-5 sample has been further heated to remove the inter-channel PY. In contrast to 1-PY, the fluorescence and phosphorescence of PY@MOF-5 can be easily recovered after heating the sample at 50 °C for 6 hours, which further confirms the guest-induced emission change in the PY@MOF-5 system. Moreover, the addition and removal of the PY molecules can be recycled by alternative treatment with PY and high-temperature conditions for at least three times, as confirmed by the reversible luminescent transformation between long/short wavelengths (Fig. 7b and c).

**Fig. 8:**
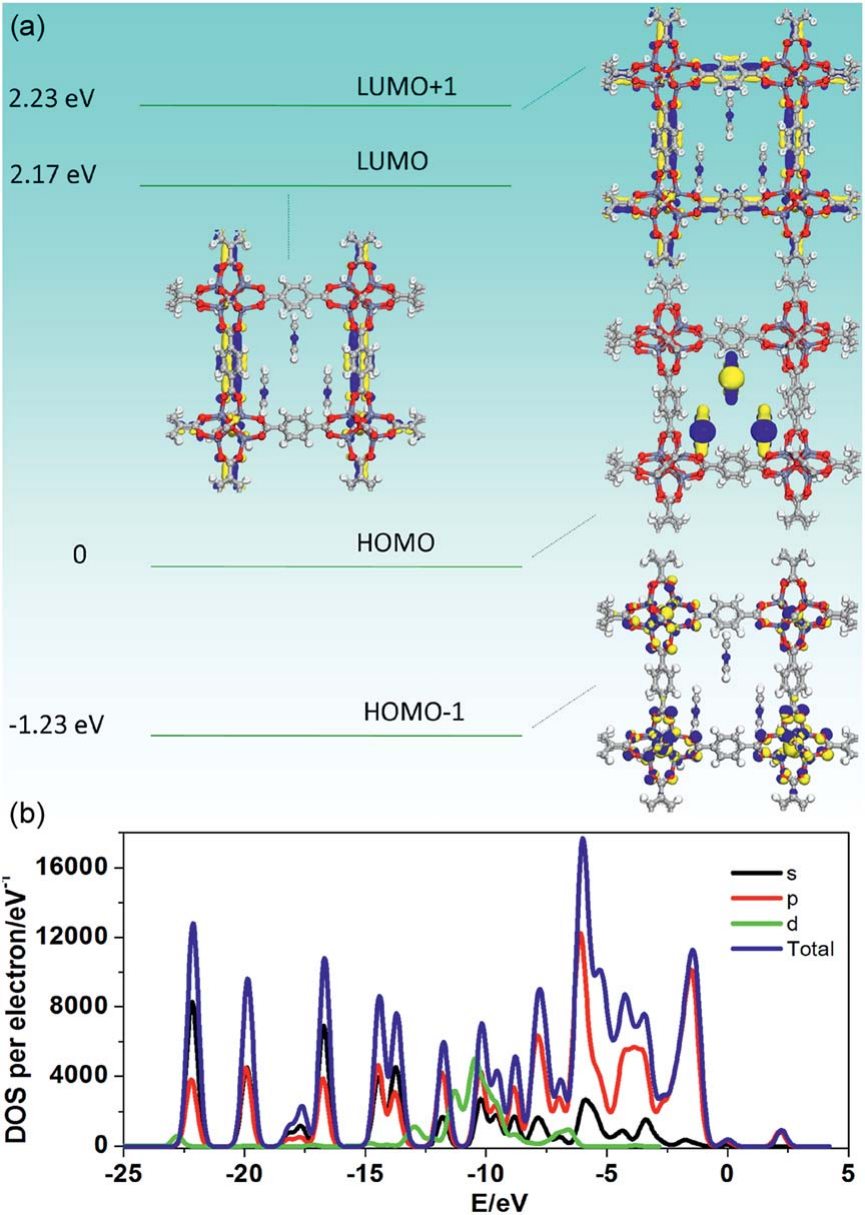
Frontier orbital profiles (a) and total/partial electronic density of state (TDOS and PDOS, (b)) for the DFT-optimized structure of PY@MOF-5 (H: white; O: red; C: gray; Zn: blue-gray; N: blue).

To further understand how the introduction of the PY guest can induce the phosphorescence change from a green to red color, PDFT was further performed on the PY@MOF-5 host–guest system, in which three PY guest molecules were selected as a model to assemble within one nanochannel of MOF-5 uniformly (Fig. 8). Energy level analysis shows that the introduction of PY highly reduced the band gap (2.17 eV), compared with pristine MOF-5 (*ca.* 3.4 eV). The HOMO is mainly contributed from the PY molecules, and the HOMO−1, LUMO, and LUMO+1 are located within the MOF-5 host framework, confirming that the PY can largely influence the frontier orbital distributions in the host–guest system due to the introduction of intermediate energy levels. Therefore, it can be concluded that the strategy based on the change in host–guest interactions as well as guest-induced structural transformation can largely tune the phosphorescence emission in a wide range.

## Conclusions

In summary, we have confirmed that MOFs can serve as one new type of RE-free and NM-free room-temperature long-afterglow photoemission material, which could potentially be used in illumination and light-emitting applications. The key to the formation of afterglow MOFs at room temperature can be related to the selection of suitable phosphor ligands and metal ions with strong coordination interactions and potential charge transfer processes. The as-prepared [Zn(TPA)(DMF)] MOF also presents a reversible temperature-sensitive phosphorescence emission. Moreover, due to the dynamic tunabilities of both structures and host–guest interactions, a wide range of phosphorescence color changes from cyan to yellow or from green to red can be achieved. Based on the selective phosphorescence wavelength shift upon treatment with guest solvents, the MOFs can serve as a potential sensor for pyridine detection. By virtue of both experimental and theoretical studies on the host–guest phosphorescence MOF systems, this study not only presents a feasible method for fabricating MOF-based afterglow materials with tunable phosphorescence, but also gives a detailed understanding on the geometric/electronic structures of the guest molecules within the MOF matrix. It can be expected that the tunable long-afterglow MOFs can be extended to other systems based on their facile design, cost-effective solution process and controllable synthesis.

## Experimental section

### Reagents and materials

Analytically pure Zn(NO_3_)_2_·6H_2_O, terephthalic acid (TPA), and *N*,*N*′-dimethylformamide (DMF) were purchased from Sigma Chemical. Co. Ltd. and used without further purification.

### Synthesis of 1-DMF

A mixture of TPA (166 mg), and Zn(NO_3_)_2_·6H_2_O (595 mg) was added to DMF (10 mL) in a 25 mL Teflon-lined stainless steel vessel. The mixture was heated at 100 °C for 6 h, and then slowly cooled down to room temperature. A large quantity of block crystals of [Zn(TPA)(DMF)] (1-DMF) were obtained, which were washed with DMF several times under ambient conditions. IR/cm^−1^ (KBr): 3428 (m, br), 2930 (m), 1653 (s), 1550 (s), 1496 (m), 1435 (m), 1386 (s), 1104 (m), 839 (s), 750 (s), 677 (m).

### Synthesis of MOF-5

TPA (166 mg) was dissolved in DMF (15 mL) in a 25 mL Teflon-lined stainless steel vessel, and the solution was sonicated for 30 min. Zn(NO_3_)_2_·6H_2_O (595 mg) was added to the above solution. The clear solution was heated at 120 °C for 6 h, and then slowly cooled down to room temperature. A large quantity of cubic block crystals of MOF-5 were obtained, which were washed with DMF several times under ambient conditions. IR/cm^−1^ (KBr): 3455 (w, br), 2957 (w), 1652 (s), 1601 (s), 1580 (s), 1388 (s), 1104 (m), 825 (w), 752 (s).

### Characterization

Single-crystal X-ray diffraction data for all compounds were collected using a Bruker SMART APEX CCD diffractometer^26^ equipped with graphite monochromatized Mo-Kα radiation (*λ* = 0.71073 Å) at room temperature using the ω-scan technique. Empirical absorption corrections were applied to the intensities using the SADABS program.^27^ The structures were solved using the SHELXS-97 program^28^ and refined using the SHELXL-97 program.^29^ All nonhydrogen atoms were refined anisotropically. The crystallographic data for 1-DMF are listed in Table S1 and S2.† Crystallographic data for 1-DMF in this paper have also been deposited with the CCDC as deposition no. 1450104. PXRD patterns of all compounds were collected using a Rigaku Ultima-IV automated diffraction system with Cu Kα radiation (*λ* = 1.5406 Å). Measurements were made in a 2*θ* range of 5–50° at room temperature with steps of 0.02° (2*θ*) and a counting time of 0.2 s per step. The operating power was 40 kV, 30 mA. IR spectra were recorded in the range of 4000–400 cm^−1^ using a Tensor 27 OPUS (Bruker) FT-IR spectrometer with KBr pellets. Thermogravimetric analysis (TGA) experiments were carried out using a Perkin-Elmer Diamond SII thermal analyzer from room temperature to 800 °C under a nitrogen atmosphere at a heating rate of 10 °C min^−1^. Photoluminescence (PL) spectra, and time-resolved PL spectra experiments were conducted using an Edinburgh FLS980 fluorescence spectrometer. The excitation source for the time-resolved lifetime measurements was a microsecond flashlamp with time-resolved single photon counting-multi-channel scaling (MCS) mode. The solid UV-vis absorption spectra were collected using a Shimadzu 440 U-3000 spectrophotometer. The PL quantum yield (PLQY) values at room temperature were estimated using a Teflon-lined integrating sphere (F-M101, Edinburgh, diameter: 150 mm and weight: 2 kg) in a FLS980 fluorescence spectrometer. The temperature dependence of the phosphorescence intensity, and phosphorescence lifetime were measured using a temperature controller attached to a cryostat (Oxford Ltd. Optistat DN2) using an FLS980 fluorescence spectrometer. Photographs of single crystals of the MOFs were taken under an OLYMPUS IXTI fluorescence microscope. Photographs of the afterglow for 1-DMF taken at different time intervals before and after turning off the UV excitation (365 nm) under ambient conditions were captured using a Canon digital camera (EOS 700D: the ISO value was 1600, time of exposure was set to an automatic mode, and the value of the aperture was 4.5).

### Electronic structure calculations of selected Zn-based MOFs

All calculations were performed using the periodic density functional theory (DFT) method using the Dmol3 (ref. 30*a* and *b*) module in the Material Studio software package.^30*c*^ The initial configuration was fully optimized by the Perdew–Wang (PW91)^30*d*^ generalized gradient approximation (GGA) method with double numerical basis sets plus a polarization function (DNP). The core electrons for the metals were treated using effective core potentials (ECP). The self-consistent field (SCF) converged criterion was within 1.0 × 10^−5^ hartree per atom and the converging criterion of the structure optimization was 1.0 × 10^−3^ hartree bohr^−1^. The Brillouin zone was sampled by 1 × 1 × 1 *k*-points, and test calculations revealed that the increase of *k*-points does not affect the results.

## Supplementary Material

SC-007-C6SC00563B-s001

SC-007-C6SC00563B-s002
