# Supplementary material for: Long-afterglow metal–organic frameworks: reversible guest-induced phosphorescence tunability
Source: Chem Sci. 2016 Mar 24;7(7):4519–26. doi: 10.1039/c6sc00563b (PMC6016333; doi:10.1039/c6sc00563b)
Supplement: SC-007-C6SC00563B-s001 [file SC-007-C6SC00563B-s001.pdf]

Electronic Supplementary Material (ESI) for Chemical Science.
This journal is © The Royal Society of Chemistry 2016

Electronic Supplementary Information

Long-afterglow Metal-Organic Frameworks: Reversible Guest-induced Phosphorescence Tunability

Xiaogang Yang[a] and Dongpeng Yan*[a,b]

[a] *Key Laboratory of Theoretical and Computational Photochemistry, Ministry of Education, College of Chemistry, Beijing Normal University, Beijing 100875 (P. R. China). E-mail: yandp@bnu.edu.cn*

[b] *State Key Laboratory of Chemical Resource Engineering, Beijing University of Chemical Technology, Beijing 100029, (P. R. China).*

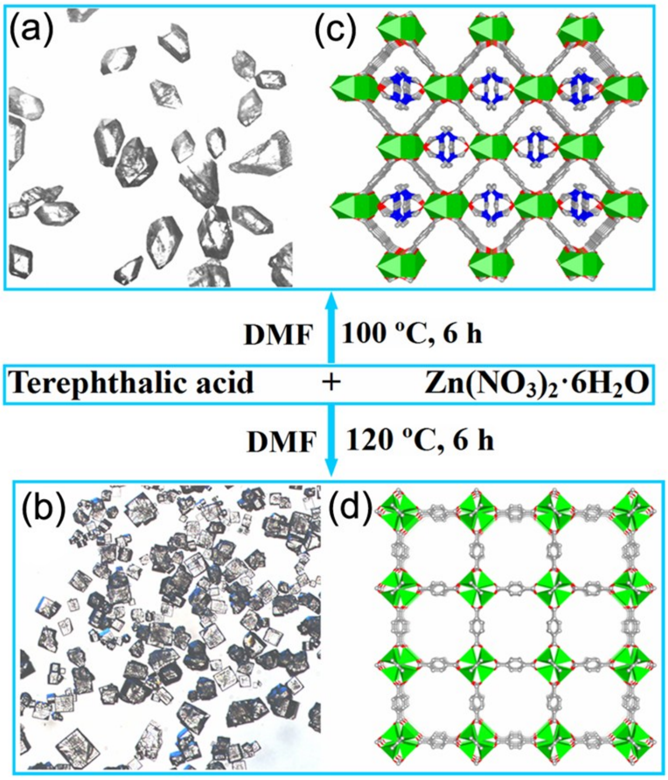

Figure S1. Scheme for synthesis of **1-DMF** (a) and **MOF-5** (b) single crystals; single crystal structure of **1-DMF** (c) and **MOF-5** (d). Hydrogen atoms have been omitted for clarity. C, grey; N, blue; O, red; Zn, green.

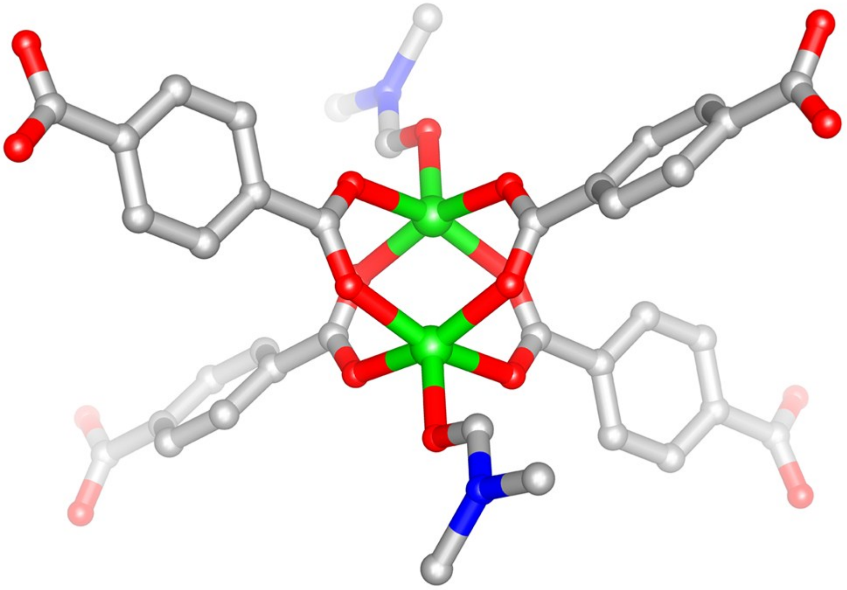

Figure S2. Ball-and-stick view of the paddle-wheel unit in **1-DMF**.

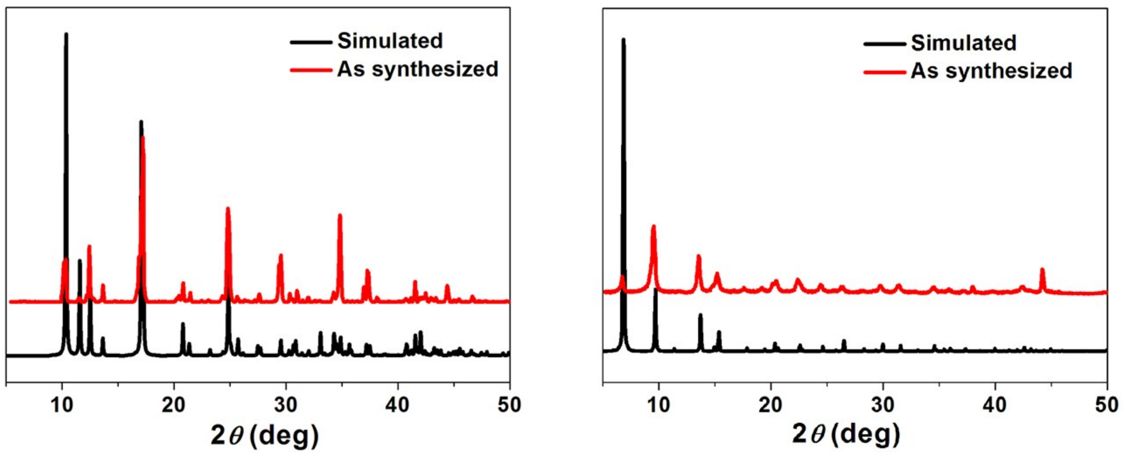

(a) (b)

Figure S3. Simulated and as synthesized PXRD patterns of **1-DMF** and **MOF-5**.

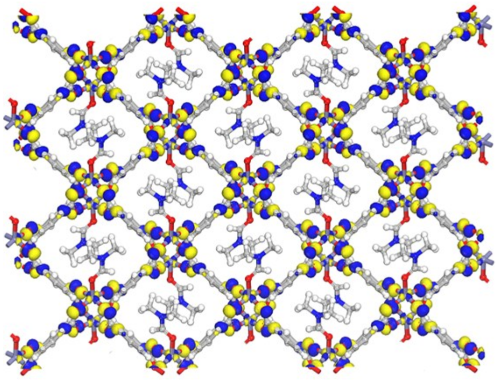

HOMO-1

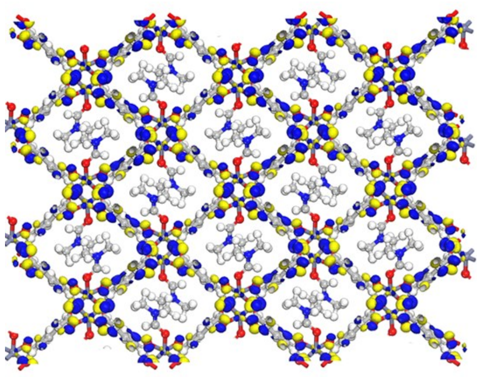

HOMO-2

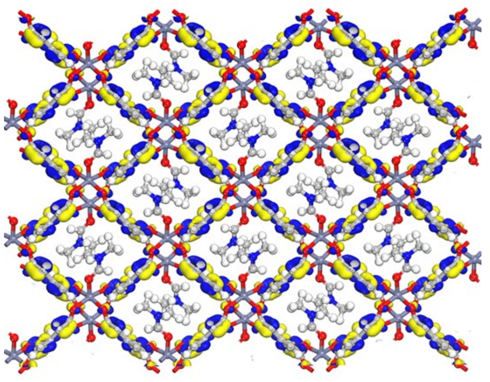

LUMO+1

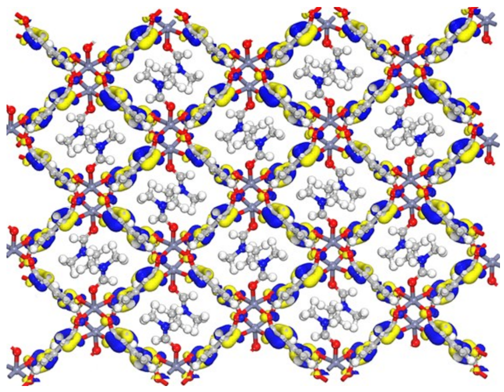

LUMO+2

Figure S4. The electron-density distribution of various orbitals involved in **1-DMF**.

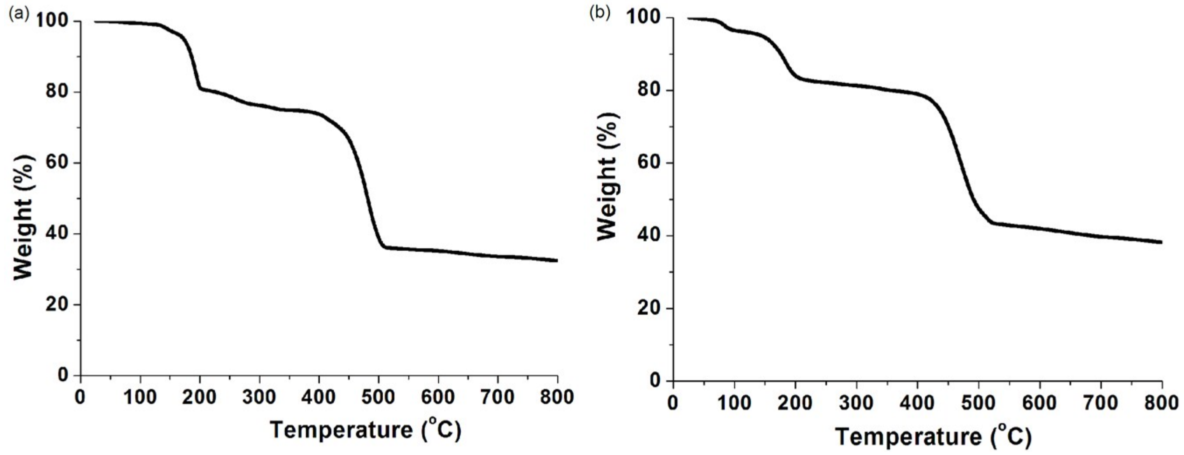

Figure S5. Thermogravimetric (TG) analysis curves of **1-DMF** (a) and **MOF-5** (b).

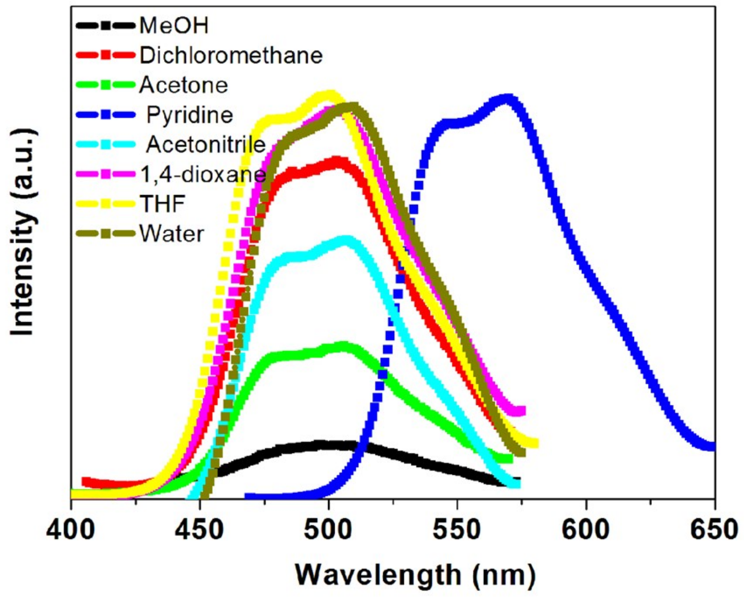

Figure S6. Phosphorescence spectra of **1-DMF** after immersing in different solvents.

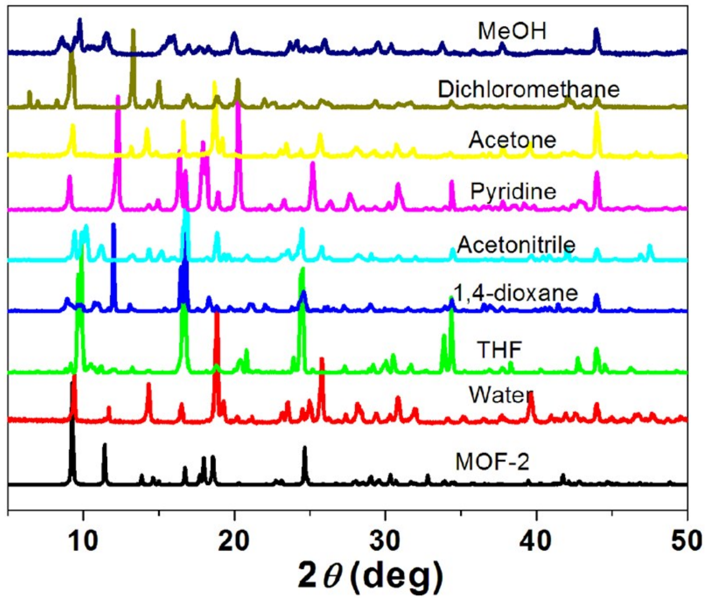

Figure S7. PXRD patterns of **MOF-2** and **1-DMF** after immersing in different solvents.

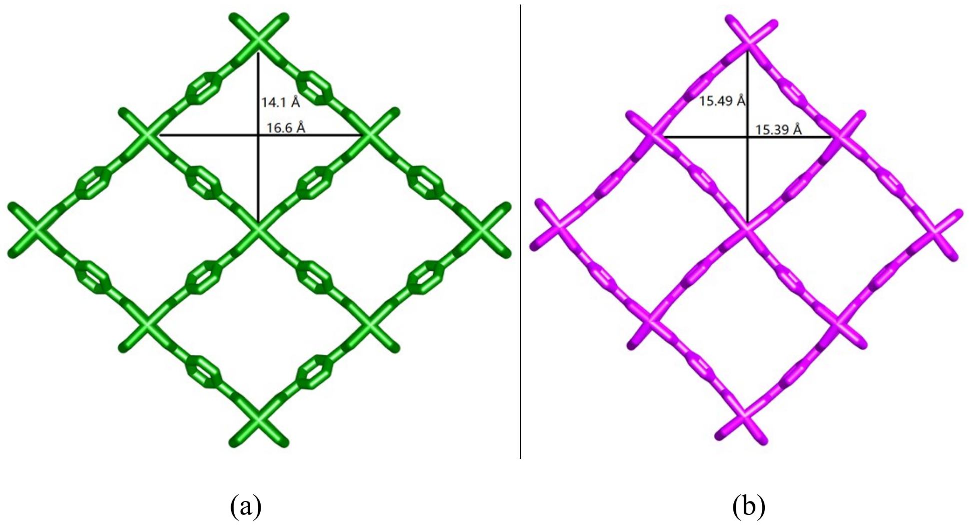

Figure S8. View of the 2D layer for **1-DMF** (a) and **MOF-2** (b).

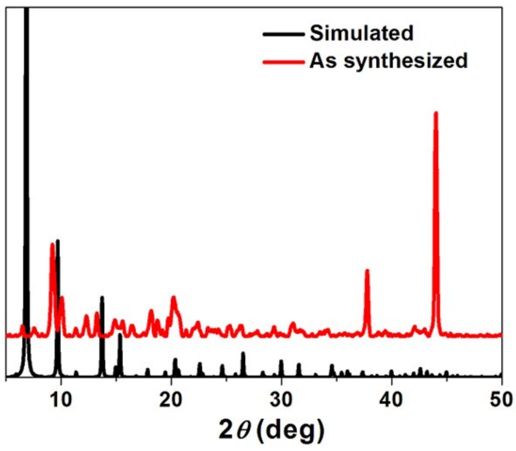

Figure S9. PXRD patterns of **MOF-5** treated with pyridine.

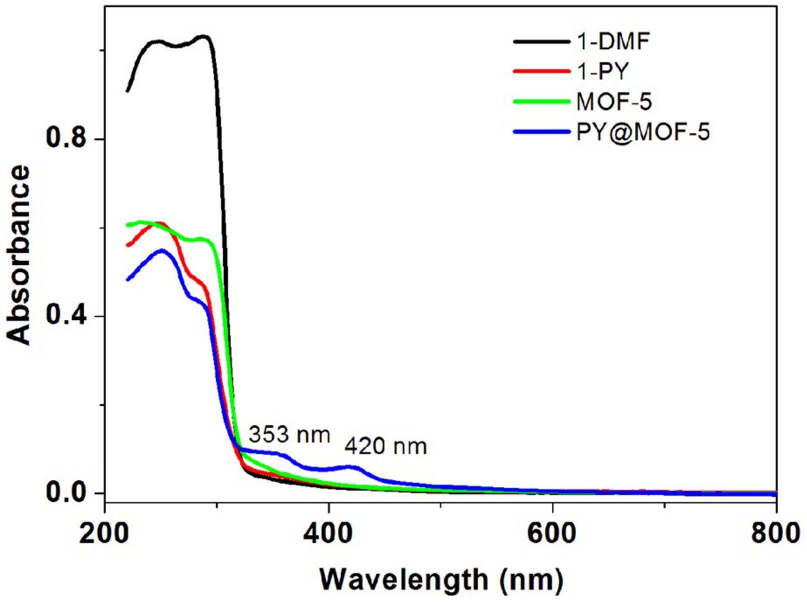

Figure S10. UV-visible absorption spectra for **1-DMF**and **MOF-5** and the samples after pyridine treatment.

Table S1. Crystallographic data for **1-DMF** [a].

Compound	**1-DMF**
Formula	$C_{11}H_6ZnNO_5$
Formula weight	297.54
Crystal system	Monoclinic
Space group	*C*2/*m*
a (Å)	11.191(6)
b (Å)	14.136(8)
c (Å)	7.975(5)
α (°)	90
β (°)	106.894(7)
γ (°)	90
V (Å^3)	1207.2(12)
Z	4
D_c (g/cm^3)	1.637
Abs. coeff. /mm^{-1}	2.045
F(000)	596
θ range for data collection (°)	2.39 / 25.49
GOF	1.236
R [$I > 2\sigma(I)$]	$R_1 = 0.0520$<br>$wR_2 = 0.1497$
R (all data)	$R_1 = 0.0532$<br>$wR_2 = 0.1501$

[a] $R= [\sum||F_0| - |F_c|| / \sum|F_0|]$, $R_W = \sum_W [|F_0^2 - Fc^2|^2 / \sum_W (|F_w|^2)^2]^{1/2}$

Table S2. Selected bond lengths (Å) and angles (°) for **1-DMF**.

Zn(1)-O(3)	1.988(7)	Zn(1)-O(2)#1	2.017(5)
Zn(1)-O(2)#2	2.017(5)	Zn(1)-O(1)	2.037(5)
Zn(1)-O(1)#3	2.037(5)		
O(3)-Zn(1)-O(2)#1	103.4(2)	O(3)-Zn(1)-O(2)#2	103.4(2)
O(2)#1-Zn(1)-O(2)#2	85.6(3)	O(3)-Zn(1)-O(1)	97.2(2)
O(2)#1-Zn(1)-O(1)	159.4(2)	O(2)#2-Zn(1)-O(1)	89.7(2)
O(3)-Zn(1)-O(1)#3	97.2(2)	O(2)#1-Zn(1)-O(1)#3	89.7(2)
O(2)#2-Zn(1)-O(1)#3	159.4(2)	O(1)-Zn(1)-O(1)#3	87.7(4)

[a]Symmetry codes for **1-DMF**: #1 -x, y, -z + 1; #2 -x, -y, -z + 1; #3 x, -y, z.

Table S3. The cell parameter comparison of **1-DMF** and **MOF-2**.

Parameter	**1-DMF**	**MOF-2**
Crystal system	Monoclinic	Monoclinic
Space group	$C2/m$	$P2_1/n$
a (Å)	11.191(6)	6.718 (3)
b (Å)	14.136(8)	15.488(7)
c (Å)	7.975(5)	12.430(8)
β (°)	106.894(7)	102.83(4)
V (Å^3)	1207.2(12)	1261.0 (11)
